# Emerging Potential Mechanism and Therapeutic Target of Ferroptosis in PDAC: A Promising Future

**DOI:** 10.3390/ijms232315031

**Published:** 2022-11-30

**Authors:** Chang Li, Xunzhe Yin, Zuojia Liu, Jin Wang

**Affiliations:** 1 State Key Laboratory of Electroanalytical Chemistry, Changchun Institute of Applied Chemistry, Chinese Academy of Sciences, Changchun 130022, China; 2 School of Clinical Medicine, Changchun University of Chinese Medicine, Changchun 130021, China; 3 Department of Chemistry and Physics, Stony Brook University, Stony Brook, NY 11794-3400, USA

**Keywords:** ferroptosis, PDAC, KRAS, tumorigenesis, therapy

## Abstract

Pancreatic cancer (PC) is a devastating malignant tumor of gastrointestinal (GI) tumors characterized by late diagnosis, low treatment success and poor prognosis. The most common pathological type of PC is pancreatic ductal adenocarcinoma (PDAC), which accounts for approximately 95% of PC. PDAC is primarily driven by the Kirsten rat sarcoma virus (KRAS) oncogene. Ferroptosis was originally described as ras-dependent cell death but is now defined as a regulated cell death caused by iron accumulation and lipid peroxidation. Recent studies have revealed that ferroptosis plays an important role in the development and therapeutic response of tumors, especially PDAC. As the non-apoptotic cell death, ferroptosis may minimize the emergence of drug resistance for clinical trials of PDAC. This article reviews what has been learned in recent years about the mechanisms of ferroptosis in PDAC, introduces the association between ferroptosis and the KRAS target, and summarizes several potential strategies that are capable of triggering ferroptosis to suppress PDAC progression.

## 1. Introduction

One of the most lethal malignancies is pancreatic cancer (PC). Pancreatic ductal adenocarcinoma (PDAC) is the most common type, which comprises more than 95% of all malignancies in PC [1]. Due to the changes in lifestyle, such as high-fat diets and alcohol abuse, the global incidence of PDAC is expected to increase in the future [2]. It is almost incurable, with a very low survival rate, poor prognosis and median patient survival of 6 months, for the reason that approximately 80–85% of patients are metastatic or unresectable. The treatments for PDAC include surgery, target therapy, chemotherapy, radiotherapy and palliative therapy [3]. PDAC is resistant to most of these therapies, the reason for which may be attributable to the fact that these treatments induce only apoptosis [4], and the dismantling of the apoptotic mechanism allows PDAC cells to acquire drug resistance.

Studies have shown that 90% of PDAC patients have Kirsten rat sarcoma virus (KRAS) mutations, and those mutations in KRAS genes can exert anti-apoptotic effects by inducing pro-apoptotic protein downregulation (e.g., BAX) and anti-apoptotic protein upregulation (e.g., Bcl-2) [5,6] in tumor cells. Based on this, bypassing the apoptotic signaling pathway to select the non-apoptotic programmed death pathway seems to be a feasible strategy for the treatment of PDAC. The development and progression of PDAC are dependent on oxidative stress and the accumulation of excessive reactive oxygen species (ROS), triggering cell death. Therefore, the survival of PDAC cells relies to a certain extent on ROS detoxification [7]. Solute carrier family 7 member 11 (SLC7A11), one of the ferroptosis regulators, is the most important transporter of cysteine, whose function is to uptake cysteine to clear the lipid ROS [8]. It has been found that the SLC7A11 is upregulated in PDAC tumor tissue [9], and the increased expression of SLC7A11 has been shown to help the growth of PDAC cells by reversing ferroptosis via upregulating the cysteine [10]. Gene expression omnibus (GEO) analysis showed that the overall survival of PDAC decreased with the upregulation of SLC7A11 expression, one of the ferroptosis regulators, in gemcitabine-resistant PDAC cells and the higher expression level of SLC7A11 in PDAC is linked to poor overall survival [11]. Based on these findings, PDAC cells are more likely to be susceptible to ferroptosis.

Ferroptosis is a type of iron-dependent non-apoptotic regulatory programmed cell death caused by excessive lipid peroxidation and subsequent plasma membrane rupture [12,13]. Meanwhile, it is also a kind of caspase-independent cell death [14]. PDAC is one of the gastrointestinal (GI) tumors. Numerous studies have shown that the induction of ferroptosis can lead to GI tumor cell death [15]. The free iron concentration in PDAC tumor cells is higher than that in normal cells [16]. The modulation of ferroptosis in tumor cells may become a new therapeutic method for PDAC.

This review will provide a summary of recent relevant advances, approaches, and defects in the ferroptosis-based treatment of PDAC from the perspectives of iron metabolism, oxidative stress, lipid peroxidation and system xc^-^/glutathione (GSH)/Glutathione peroxidase 4 (GPX4) axis.

## 2. The Core Mechanism of Ferroptosis

In 2012, Dixony first proposed the concept of ferroptosis to define a type of iron-dependent non-apoptotic cell death in cancer cells with KRAS mutations [17].

**Figure 1:**
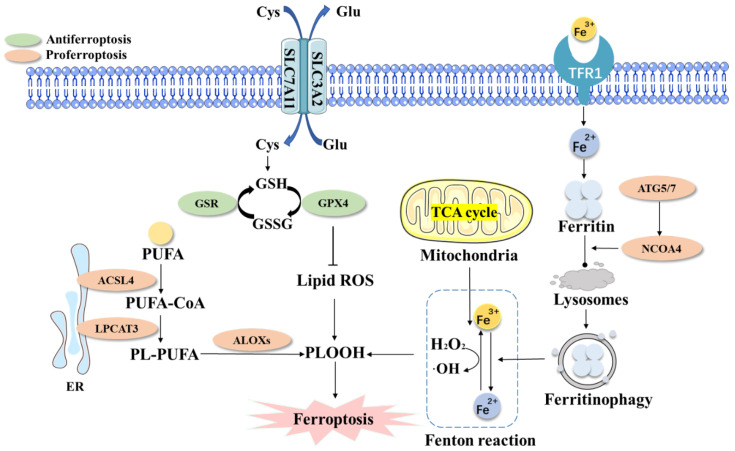
The core mechanism of ferroptosis. Ferroptosis is mainly caused by iron-dependent lipid peroxidation (PLOOH). The xc^-^ system imports cystine into cells with the 1:1 counter-transport of glutamate. GPX4 converts GSH to GSSG and reduces PLOOH, thereby blocking the lipid peroxidation chain reaction and inhibiting ferroptosis. Several proteins (including TFR1, NCOA4, ATG5, and ATG7) mediate ferritinophagy and promote iron accumulation in ferroptosis through the regulation of iron metabolism. Fe^2+^ can greatly induce PLOOH through the Fenton reaction, which then induces ferroptosis. Increasing PUFA synthesis can increase the sensitivity to ferroptosis, which is mainly regulated by ACSL4 and LPCAT3. Abbreviations: SLC7A11,zd solute carrier family 7 member 11; SLC3A2, solute carrier family 3 member 2; GSH, glutathione; GSSG, glutathione disulfide; GPX4, glutathione peroxidase 4; PLOOH, phospholipid hydroperoxide; ROS, reactive oxygen species; H_2_O_2_, hydrogen peroxide; Fe^2+^, ferrous iron; Fe^3+^, ferric iron; TFR1, transferrin receptor 1; NCOA4, nuclear receptor coactivator 4; ATG5, autophagy-related 5; ATG7, autophagy-related 7; PUFAs, polyunsaturated fatty acids; PUFA-CoA, polyunsaturated fatty acyl-coenzyme a; PL-PUFA, polyunsaturated phospholipid; ALOX, lipoxygenase; LPCAT3, lysophosphatidylcholine acyltransferase 3; ACSL4, acyl coenzyme A (CoA) synthetase long-chain family member 4; ER, endoplasmic reticulum.

Ferroptosis can be induced through extrinsic and intrinsic pathways (Figure 1) [18]. The extrinsic pathway is mainly induced by the suppression of the system xc^-^-GSH-GPX4 axis [19]. System xc^-^ is a transmembrane protein that is composed of SLC7A11 and solute carrier family 3 member 2 (SLC3A2) across the cell membrane to mediate the exchange of extracellular cystine and intracellular glutamate in a 1:1 ratio [20]. GSH acts as a substrate for the antioxidant GPX4 to decrease toxic lipids ROS during ferroptosis. GPX4 is a type of intracellular antioxidant enzyme that catalyzes the conversion of GSH into oxidized glutathione (GSSG) and converts phospholipid hydroperoxides (PLOOH) into corresponding phospholipid alcohols [21], thereby inhibiting lipid peroxidation and preventing ferroptosis [22]. Polyunsaturated fatty acids (PUFAs) are the main peroxidation substrates for ferroptosis in cell membranes [23]. The increased synthesis of PUFAs can improve susceptibility to ferroptosis, which is positively regulated by acyl coenzyme A (CoA) synthetase long-chain family member 4 (ACSL4) [24]. 

The intrinsic pathway of ferroptosis can be induced by many small molecular compounds [25]. The inducers mainly include erastin [17], Sorafenib [26], R3L3 [27] and so on (Table 1). The inhibitors include Ferrostain-1 [28], Lipoxstrain-1 [29], N-Acetylcysteine [30] and so on (Table 2). Autophagy can trigger ferroptosis [31] by leading to the degradation of the intrinsic iron storage protein ferritin or degrading solute carrier family 40 member 1 (SLC40A1) to limit the intracellular iron exporter [32]. SLC40A1 was a new autophagic substrate, contributing to ferroptosis resistance. Autophagy is required for SLC40A1 degradation during ferroptotic cancer cell death. The study confirmed that the downregulation of the SLC40A1 protein could be reversed by knocking down SQSTM1, which is the critical autophagy receptor. Immunoprecipitation and RNAi experiments identify that SQSTM1 can recognize and drive the degradation of SLC40A1 during ferroptosis [32] by preventing iron efflux and leading to iron accumulation in cells. In recent years, a large number of basic studies have confirmed that ferroptosis plays an important regulatory role in a variety of diseases, and studies have found that tumor cells are more sensitive to intracellular iron than normal cells [33,34].

## 3. KRAS of Ferroptosis in PDAC

The characteristics of cancer are described as inflammation and metabolic adaptations and are related to tumor development and malignant progression [38]. Chronic inflammation and inflammatory mediators play key roles in the stages of PDAC [39]. 

The mutagenesis, accumulation of oncogenes and tumor suppressor genes can be accelerated by the sustained generation of inflammatory mediators [40]. The inflammation leads acinar cells to KRAS-mediated transformation by the activation of the mitogen-activated protein kinase (MAPK) pathway [41]. Mutant KRAS upregulates MYC via the MAPK pathway, resulting in increasing rate-limiting glycolytic enzymes, which sustain elevated nucleotide biosynthesis [42].

**Figure 2:**
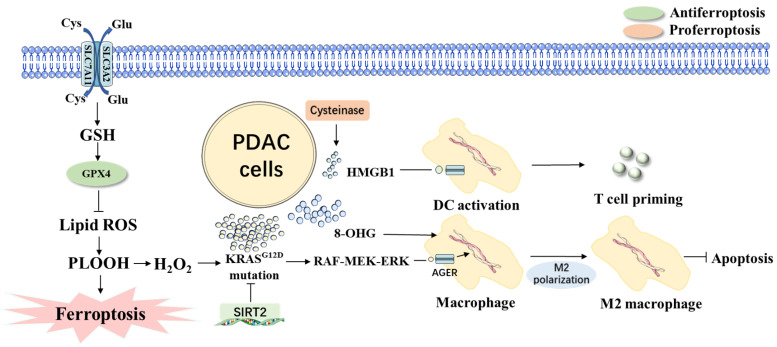
KRAS of ferroptosis in PDAC. The depletion of GPX4 or the release of H_2_O_2_ from PLOOH causes the release of 8-OHG, which leads to the activation of cytokine production in macrophages and promotes tumor growth. Ferroptotic PDAC cells can also release the KRAS^G12D^ protein, activating the RAS-RAF-MEK-ERK pathway, thereby inducing AGER-dependent macrophage M2 polarization and producing immunosuppressive cytokines in the tumor microenvironment and inhibiting apoptosis from promoting tumor cell growth. By contrast, the depletion of SLC7A11 in PDAC cells or the administration of cyst(e)inase suppresses tumor growth by releasing HMGB1 to initiate T cells and thus generate antigen-specific adaptive immune responses. Abbreviations: 8-OHG, 8-hydroxy-2′-deoxyguanosine; AGER, advanced glycosylation end product-specific receptor; GPX4, glutathione peroxidase 4; GSH, glutathione; H_2_O_2_, hydrogen peroxide; HMGB1, high mobility group box 1; PDAC, pancreatic ductal adenocarcinoma; SLC7A11, solute carrier family 7 member 11; PLOOH, phospholipid hydroperoxide.

Like inflammation, metabolic reprogramming is described as one of the characteristics of cancer [43]. Several studies have characterized one of the key events in PDAC as metabolic reprogramming [44]. KRAS mutations mediate the noncanonical reprogramming, which can elevate the levels of glucose and glycolytic intermediates, cellular redox potential, fatty acids, and glutamine uptake that provide nearly perfect conditions for cancer cells to thrive (Figure 2) [45,46].

### 3.1. KRAS of Ferroptosis in PDAC

Ferroptosis was originally described as a type of cell death specifically occurring in cancer cells with RAS mutations and characterized by iron-dependent lipid peroxidation [2], as the activation of oncogenic RAS-RAF-MEK-ERK signaling facilitates the production of ROS, thereby sensitizing cells to ferroptosis [47].

The KRAS gene is a member of the RAS gene family. KRAS can bind to GTPs, then hydrolyze them and terminate the cellular pathway signaling. The KRAS gene is an important molecular switch in the cell [48]. Genomics has demonstrated that KRAS mutations are the most common type of mutations in PDAC patients, often occurring at an early stage of tumor mutation and with a mutation rate as high as 90% [49]. The overall survival rate of PDAC patients with KRAS mutations is significantly lower than other cancer patients, and the most common mutation codon is the 12 mutation [50]. Forty-four percent of KRAS mutations in PDAC are G12D mutations, 20% are G12V mutations, and 1% are G12C mutations [51,52]. Mutation of the KRAS gene causes the KRAS protein to lose its role as a molecular switch which causes GTP hydrolysis to be impaired, and the KRAS protein remains in an activated state affecting cell signaling pathway transduction, activating cell transformation, proliferation, invasion and survival and causing cell carcinogenesis [53]. It was found that genetically engineered mice with KRAS mutations developed pancreatic intraepithelial neoplasia (PanIN) and eventually developed PDAC [54]. The RAS-RAF-MEK-ERK signaling cascade is a well-studied and well-defined RAS downstream signaling pathway and is the most important oncogenic pathway in human cancers. Through the study of the KRAS downstream signaling pathway, selective activation of the RAS-RAF-MEK-ERK signaling pathway can induce PanIN and pancreatic cancer development, and the RAS-RAF-MEK-ERK signaling cascade is the key to PDAC development, maintenance and treatment [55].

KRAS mutation promotes the proliferation, metastasis and invasion of pancreatic cancer cells and affects the tumor microenvironment (TME) and metabolic reprogramming [56]. Changes in TME are important histopathological markers of PDAC progression [57]. Ferroptosis is a regulated programmed cell death (PCD) associated with the release of damage-associated molecular patterns (DAMPs). DAMPs are released by dead cells and act as mediators of inflammatory and immune responses that regulate TME [58]. It was shown that the ferroptosis-mediated release of DAMPs could drive macrophage polarization in the TME of PDAC cells and promote tumor cell growth, suggesting a potential pathological role of ferroptosis in cancer development [59].

Selective induction of ferroptosis in tumor cells enhances the effect of chemotherapy on tumor cells [60]. Ferroptosis has a crucial role in the development of PDAC. PDAC has cysteine-dependent properties. PDAC cells can improve tumor cell survival by reducing their own oxidative stress through the synthesis of GSH, thus inhibiting ferroptosis levels [10]. In PDAC, aspartate aminotransferase (GOT1) acts as a negative regulator by maintaining oxidative stress levels and inhibiting ferroptosis, thereby promoting PDAC cell proliferation for tumor growth [61]. The ferroptosis-negative regulator solute carrier family 2 member 6 (SLC2A6) inhibits ferroptosis in PDAC cells by activating the nuclear factor erythroid 2-related factor 2 (NRF2) pathway [62].

It was found that the deletion of GPX4 and SLC7A11 genes in the pancreas accelerates pancreatitis as well as pancreatic tumorigenesis in KRAS^G12D+^ genetically engineered mice, a behavior which can be reversed by the ferroptosis inhibitor liprostatin-1 [63]. Ferroptotic PDAC cells can release KRAS^G12D^ protein through autophagy leading to the polarization of macrophages toward the pro-tumor M2 phenotype (Figure 2) [59]. This finding reinforces that cell death-induced inflammation-associated immunosuppression can accelerate KRAS-driven PDAC tumorigenesis. Therefore, most PDACs should be sensitive to ferroptosis activators in theory [64].

Ferroptotic damage creates an inflammatory tumor microenvironment for tumor growth and development, which can release DAMPs. Researches show that the depletion of GPX4 in the pancreas or a high-iron diet can accelerate the development of KRAS^G12D^-driven pancreatic tumors in mice [65]. This process is mediated by the release of 8-hydroxy-2′-deoxyguanosine (8-OHG) in ferroptotic cells (Figure 2) [66]. The released 8-OHG induces the release of cytokines to maintain the chronic inflammatory microenvironment of pancreatic tumorigenesis driven by KRAS. In contrast, the KRAS^G12D^ mutation-driven pancreatic tumors in mice can be inhibited by the deletion of SLC7A11 in the pancreas, and this result suggested that additional P53 mutations may transform the carcinogenic effects of ferroptotic damage into anticancer effects in KRAS^G12D^-driven PDAC [10]. These results explain the basic aspects of the inflammatory tumor microenvironment mediated by ferroptotic death in PDAC.

### 3.2. Targeting KRAS to Cure PDAC Based on Ferroptosis

Mutant KRAS proteins are similar to normal proteins, but the lack of deep pockets for small molecule inhibitors makes specific treatment difficult. With the understanding of KRAS mutations, KRAS type G12C has been successfully targeted, and sotorasib is the first targeted covalent inhibitor that directly targets KRAS mutant proteins. Sotorasib inhibits the activation of KRAS proteins by covalently interacting with newly-generated cysteine residues of G12C mutants to achieve cancer suppression [67]. MRTX849 is another highly selective covalent inhibitor targeting the G12C mutant, with the advantage of a 1000-fold increase in target selectivity compared to sotorasib [68].

RAS-RAF-MEK-ERK, a downstream pathway of RAS, has emerged as a new target for pancreatic cancer therapy. It was found that ferroptosis was associated with the RAS-RAF-MEK-ERK pathway. Lipid peroxidation is one of the markers of ferroptosis, and the RAS-RAF-MEK-ERK pathway was found to be one of the regulators of lipid peroxidation [69]. In PDAC cells, ferroptosis inducers inhibit the tumor microenvironment by activating the RAS-RAF-MEK-ERK pathway to treat pancreatic cancer [70]. Basic research has found Erastin induces the activation of signal transduction and activator of transcription 3 (STAT3) in ferroptosis. STAT3-mediated induction of cytosolic lysosomal death by cathepsin B expression in PDAC is an important pathway of ferroptosis [70]. And the RAS-RAF-MEK-ERK pathway is required for erastin-induced STAT3 activation and subsequent ferroptosis in PDAC cells. The reason probably is that ERK is the upstream kinase for STAT3 activation in response to various stressors [71,72,73]. The function between mutant and wild-type RAS in controlling STAT3-activated ferroptosis remains to be further explored, and targeting STAT3-dependent ferroptosis may represent a novel opportunity for the treatment of PDAC.

Research suggests a link between tumor microenvironment and ferroptosis-related gene expression in pancreatic cancer patients. Ferroptosis cancer cells can release high-mobility group protein box 1 (HMGB1) in an autophagy-dependent manner (Figure 2) [74]. HMGB1 belongs to a family of intracellular molecules called DAMPs. Once released outside the cell, they acquire immune-stimulating properties and function as adjuvants that promote the activation of innate and adaptive immune systems by binding to pattern-recognition receptors, suggesting that ferroptotic cancer cells may be immunogenic [75]. PDAC cells release KARS^G12D^ exosomes in the form of autophagy-dependent ferroptosis, which is phagocytosed by macrophages and induces M2 polarization to inhibit its phagocytosis [59].

Induce ferroptosis may be a new treatment strategy to cure PDAC because it can improve the sensitivity of PDAC. The study of the role of the ferroptosis pathway in PDAC by targeting RAS proteins has important clinical implications for enhancing the efficacy of tumor treatment in PDAC patients.

## 4. Ferroptosis in PDAC Tumorigenesis

### 4.1. Link between Genes and Ferroptosis Involved in PDAC

Sirtuin 2 (SIRT2) is a member of the sirtuin family that is associated with many biological processes, such as the cell cycle, metabolic homeostasis and tumorigenesis. A recent study has shown that KRAS mutations can be induced by SIRT2 loss. KRASK147 is a novel SIRT2-specific deacetylation target for KRAS, which can regulate its activity and ultimately influence tumor growth [76]. A recent study on PDAC in mice showed that the lack of SIRT2 increased the pancreatitis-permissive phenotype, exhibited extensive tissue fibrosis and delayed pancreatic tissue recovery [77]. Importantly, the SIRT2 deficiency induced an inflammatory phenotype permissiveness for the accumulation of KRAS mutation cells. This is closely related to the development of PDAC.

**Figure 3:**
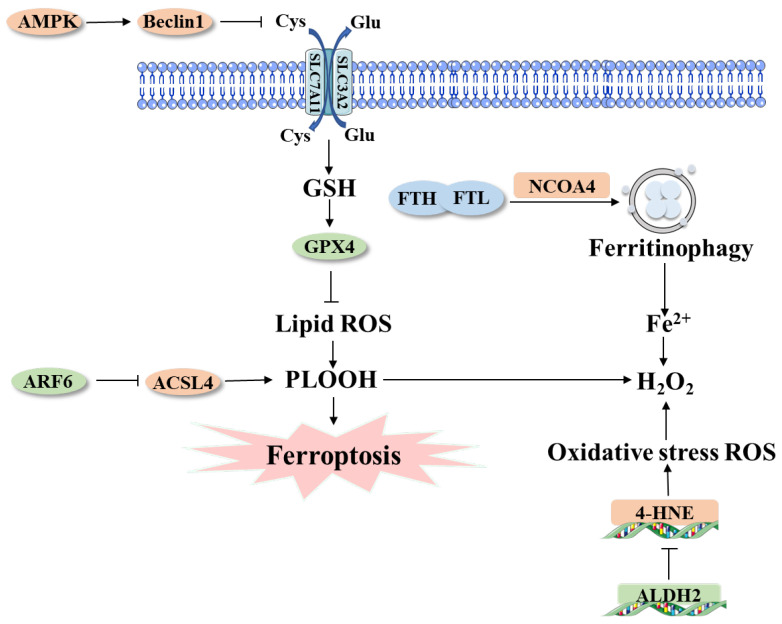
Mechanism of ferroptosis involved in PDAC. Ferroptosis is an iron-dependent cell death driven by lipid peroxidation. There are multiple ways to regulate the level of ferroptosis in PDAC cells, such as transcription factors, autophagic degradation and metabolic pathways. System xc^-^ and GPX4 act as the key regulators in the process of ferroptosis in PDAC. AMPK-Beclin1 pathway trigger ferroptosis by inhibiting system xc^-^. Irisin serves as a suppressor of ferroptosis by increasing the accumulation of lipid reactive oxygen and the depletion of GSH. NCOA4 accumulates in lysosomes and then promotes ferritinophagy, leading to iron accumulation and, ultimately, ferroptosis. ARF6 increases the expression of ASCL4 and induces ferroptosis by increasing the expression of PLOOH. ALDH2 decreases 4-HNE production in PDAC cells and blocks the ferroptosis pathways by reducing oxidative stress ROS. Abbreviations: GSH, glutathione; GPX4, glutathione peroxidase 4; PDAC, pancreatic ductal adenocarcinoma; AMPK, AMP-activated protein kinase; NCOA4, nuclear receptor coactivator 4; ACSL4, acyl coenzyme A (CoA) synthetase long-chain family member 4; ALDH2, aldehyde dehydrogenase 2; 4-HNE, 4-hydroxy-2-nonenal; ROS, reactive oxygen species.

Aldehyde dehydrogenase 2 (ALDH2) is an important mitochondrial enzyme with the function of governing ethanol metabolism (Figure 3). Recent evidence suggested that the ALDH2 gene may be correlated with many cancer developments, such as pancreatic cancer, breast cancer and lung cancer. By analyzing peripheral blood leukocytes between PDAC individuals and non-cancer controls, researchers uncovered that the ALDH2 gene mutants showed a strong association with PDAC [78]. Meanwhile, serum ferritin levels were lower in ALDH2 carriers, suggesting that ALDH2 is correlated with ferritin concentrations and has the function of regulating iron metabolism and thus promoting ferroptosis [79]. In addition, ALDH2 lowers 4-hydroxynonenal (4-HNE) concentration. 4-HNE, a metabolite of lipid peroxidation, was found to have a strong association with ferroptosis (Figure 3). 4-HNE levels were relatively low in cancer tissues compared to normal tissues, and lower 4-HNE levels were also closely associated with higher ferroptosis sensitivity in tumors [80]. More malignant tumors, such as pancreatic cancer, are sensitive to ferroptosis and often exhibit a high propensity for metastasis and drug resistance, suggesting a potential anti-tumor effect of 4-HNE [81].

Precision therapy targeting genes related to ferroptosis may be a potential therapeutic approach to improve ferroptosis sensitivity and induce cancer cell death in tumor patients.

### 4.2. Other Factors in Ferroptosis Involved in PDAC

#### 4.2.1. Iron Accumulation & PDAC

Iron is necessary to maintain cell metabolism [82]. Iron accumulation plays a key role in ferroptosis and is closely related to the growth and development of tumors [83].

Autophagy is the degradation system that can regulate cellular homeostasis [84]. Autophagy-related genes are closely related to ferroptosis and play an important role in PDAC cells. Recent studies have shown that in PDAC cells, the accumulation of free iron is increased by the autophagy degradation of iron storage protein ferritin. This process is called ferritinophagy [31]. Nuclear receptor coactivator 4 (NCOA4) acts as an autophagy receptor to recognize and degrade ferritin during ferroptosis. A study proved that the inhibition of autophagy-related gene 5 (ATG5) and autophagy-related gene 7 (ATG7) in mice could significantly reduce the ferrous iron and malondialdehyde levels in cells, finally preventing the death of PDAC cell lines (PANC1 and PANC2) [31]. The study further proved that NCOA4 is the receptor of ATG5 and ATG7 that mediates ferritin degradation and releases ferrous iron. The induction of autophagy-dependent ferroptosis may provide a way to kill PDAC cells, and the ATG5-ATG7-NCOA4 autophagy pathway is a new target to treat pancreatic cancer (Figure 3) [85].

#### 4.2.2. Oxidative Stress & PDAC

Unregulated proliferation is a major hallmark of cancer, and the production of ROS is inevitable during this process. The major characteristic of ferroptosis is the accumulation of lipid ROS [86]. Although ROS can promote tumorigenesis, an over the level of ROS may be toxic and inhibit tumor growth. The activation of oxidative stress and weakening of the antioxidant barrier causes quantitative ROS production and subsequent ferroptosis in cancer cells [61]. Recent studies have shown that in PDAC cells, the induction of ferroptosis inhibited PDAC cell growth successfully [10]. A study with genetically engineered mice has found that the deletion of SLC7A11 can induce tumor-selective ferroptosis and the inhibition of PDAC growth. Cancer-associated fibroblasts (CAFs) are highly dependent on SLC7A11, and the removal of SLC7A11 increased ferroptosis of CAFs in PDAC [87]. 

#### 4.2.3. Lipid Peroxidation & PDAC

Lipid peroxidation is the main feature of ferroptosis, and it is mainly caused by PUFAs under the action of lipoxygenase and ROS, which eventually activate ferroptosis and inhibits tumor development [88].

As a member of the RAS family, adenosine diphosphate (ADP)-ribosylated factor 6 (ARF6) is closely related to autophagy and immunity and plays a role in regulating the invasion, metastasis and proliferation of cancer cells (Figure 3) [89]. Studies have shown that ARF6 is highly expressed in pancreatic cancer cell lines PANC1 and PANC2, and the inhibition of ARF6 can promote RSL3-induced ferroptosis. Further experiments confirmed that abrogation of ARF6 can induce lipid peroxidation and inhibit pancreatic cancer cell growth by regulating ACSL4 protein levels [90].

Irisin, a PGC-1α protein secreted by exercised muscle, is closely correlated with the induction of cellular autophagy [91]. Recent studies have shown that Irisin significantly increased ferroptosis in PDAC cells and that Irisin’s action on PDAC cells resulted in significant upregulation of the lipid peroxidation levels and the total ROS levels in PDAC cells. Conversely, when siRNA expressed by Irisin was inhibited, PDAC cell viability and relative GSH concentrations were increased, while accumulated lipid peroxidation, malondialdehyde (MDA), and Fe^2+^ levels were decreased [92]. Irisin induces ferroptosis in pancreatic cancer cells by increasing the depletion of GSH and the accumulation of lipid peroxides.

#### 4.2.4. System xc^-^/GSH/GPX4 Axis & PDAC

Ferroptosis is regulated by the system xc^-^/GSH/GPX4 axis [93]. Sorafenib is an inhibitor of the system xc^-^ and a first-line agent for the induction of ferroptosis in the treatment of cancers [94]. In the study of PDAC, sorafenib enhanced anticancer activity by inhibiting STAT3 activity from promoting ferroptosis in tumor cells [95,96]. Beclin1 is a class III phosphatidylinositol 3-kinase (PtdIns3K) complex that plays an important role in tumor formation and metastasis (Figure 3). It has been shown that Beclin1 is a key regulator of autophagy and ferroptosis, and Beclin1 can form Beclin1-SLC7A11 complex with SLC7A11, the core component of the xc^-^ system, to inhibit the activity of the xc^-^ system and thus activate ferroptosis, which is triggered by the energy metabolism center AMPK [97,98]. Beclin1 increases lipid peroxidation and GSH depletion through this mechanism, thereby promoting PDAC ferroptosis. Targeting the xc^-^ system/GSH/GPX4 axis is a potential new research direction for PDAC treatment.

## 5. Approaches Specifically Targeting Ferroptosis in PDAC Treatment

**Figure 4:**
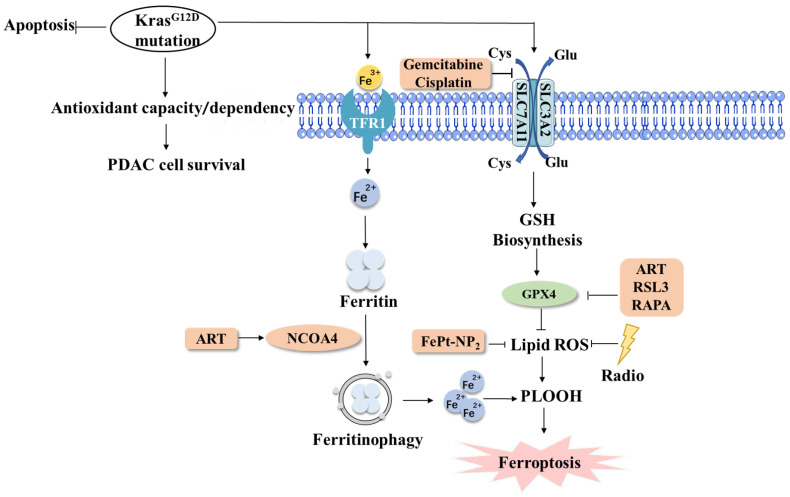
Ferroptosis in the treatment of PDAC. Kras^G12D^ mutations can inhibit PDAC cell apoptosis by blocking ferroptosis. There are many therapeutic ways to cure PDAC by inducing ferroptosis in PDAC cells, such as targeted therapy, immunotherapy, nanomedicine, and radiotherapy. Abbreviations: PDAC, pancreatic ductal adenocarcinoma.

The role of ferroptosis in PDAC tumors has become prominent, and studies on the treatment of PDAC are increasing. The induction of ferroptosis may be a new strategy to treat PDAC (Figure 4).

### 5.1. Ferroptosis & Targeted Therapy of PDAC

With the increasing research on ferroptosis regulation, ferroptosis-inducing reagents (FINs) are becoming increasingly important for tumor therapy. GSH plays a key role in ferroptosis and is considered an important target for anticancer therapy. Targeting GSH or the regulator factors that affect GSH may provide a new direction for PDAC treatment. Some studies have found that inhibiting SLC7A11 expression can induce ferroptosis by decreasing the GSH content and inhibiting the proliferation and survival of PDAC cells [99]. GPX4 is also a key factor in regulating the ferroptosis process. Autophagy inducers rapamycin (RAPA) and RSL3 cause the degradation of GPX4 protein to enhance anticancer activity in PDAC cells [100]. The antimalarial drug artesunate (ART) is a newly discovered FIN that can inhibit the activation of PDAC. The mechanism is depleting GSH and producing large amounts of PUFA via endogenous Fe^2+^ to induce ferroptosis [101]. 

The results proved that induced ferroptosis could target PDAC cells and provide new ideas for PDAC therapy. Due to the prevalence of ferroptosis sensitivity in tumor cells, FINS has been approved by the FDA as a tumor ferroptosis inducer for adjuvant therapy but is facing issues such as pharmacokinetics and specificity optimization.

### 5.2. Ferroptosis & Immunotherapy of PDAC

Immune cells, as one of the components of the tumor microenvironment, are mutually regulated with tumor cells in tumor development. Chemotherapy can stop the proliferation of cancer cells and can also act directly or indirectly on immune cells and change the TME, which is one of the common means of treating malignant tumors. TME is a hot research topic, and in cancer treatment, it is favorable to choose immunotherapy [9]. Cancer immunotherapy can restore or activate the function of cytotoxic T cells through the perforin-granzyme and Fas/Fas ligand pathways, resulting in inducing tumor cell death [102,103,104]. It has been investigated that immunotherapy-activated cytotoxic T cells enhance ferroptosis-specific lipid peroxidation in tumor cells and that increased ferroptosis contributes to the antitumor efficacy of immunotherapy. Mechanistically, cytotoxic T cells release interferon-gamma (IFNγ) to activate the JAK-STAT1 pathway and inhibit the uptake of cystine in tumor cells due to the downregulation of the System xc^-^ expression [105].

At present, the chemotherapy drugs for PDAC are mainly gemcitabine, which not only can act directly on tumor cells but also indirectly affect immune function, such as changing the function of immune cells and acting on immunosuppressive pathways [106]. Studies have shown that oxidation products released by ferroptosis cancer cells may modulate anti-tumor immunity. The molecular mechanisms related to the inhibition of ferroptosis can effectively alleviate the issue of tumor cell resistance to chemotherapeutic agents. Cisplatin-induced ferroptosis in cisplatin-resistant human head and neck cancer HNC cells by inhibiting system xc^-^ or in the absence of cysteine or glutamine excess, and increased the killing ability of cisplatin on cisplatin-resistant HNC cells [107]. Chemotherapeutic drugs can act on the tumor microenvironment, and the induction of ferroptosis can significantly improve the efficacy of chemotherapeutic agents. The related mechanism is not completely clear, and further basic research is needed to verify the relationship to optimize clinical chemotherapy regimens.

### 5.3. Ferroptosis & Nanomedicine of PDAC

In order to further optimize the efficacy and safety of anticancer drugs, nanomedicine has become a new application direction of ferroptosis [108].

Nanomedicines are drugs packaged in nanoparticles with diameters of 10–200 nm, using the enhanced permeability and retention effect (EPR) to precisely release carrier drug molecules in the TME of cancer cells to target cancer cells [109]. FePt-NP2, an iron oxide nanoparticle, can increase iron and ROS levels, and studies have shown that loading cisplatin prodrugs onto FePt-NP2 can significantly increase the sensitivity of cancer cells to cisplatin and reduce cisplatin resistance [110]. The most characteristic feature of the pancreatic cancer TME is that it has a very high degree of stromal fibrosis and a highly hypoxic stromal environment. This TME not only blocks the delivery of conventional chemotherapeutic agents but also reduces the exposure of tumor antigens, resulting in the poor efficacy of targeted drugs [111,112]. It has been shown that PDAC cells can be stably targeted by preparing Erastin [113] as well as GPX4 inhibitors [114] in nanodrug form. Nanomedicine may also reduce the toxicity of drugs to normal cells. By preparing the ferroptosis inducer IKE as nanoparticles for application in diffuse large B-cell lymphoma, reduced IKE toxicity and the ability to promote tumor suppression were observed [111].

However, due to the differences among animal models and clinical practice, as well as the different carrier types, surface charge distribution and surface chemical reactions of nanomedicines, nanomedicines are difficult to mass-produce.

### 5.4. Ferroptosis & Radiotherapy of PDAC

In the multimodal treatment of pancreatic cancer, radiotherapy remains a critical component [115]. Radiation therapy uses high-energy ionizing radiation to break the DNA double-strand of tumor cells and induce cell cycle arrest, thus causing cell death. But some tumor patients are resistant to radiation therapy.

The appropriate intensity of radiation can promote ferroptosis of tumor cells and reduce tumor growth, while ferroptosis inhibitors can reduce the efficacy of radiotherapy for tumors [116]. Erastin has been found to induce ferroptosis, enhance the sensitivity of breast, cervical, and lung cancer cells to radiation and promote cell death [117]. Through in vivo and in vitro experiments, it was found that radiotherapy can cause tumor cells to produce large amounts of lipid ROS and ACSL4, leading to lipid peroxide accumulation and inducing ferroptosis. Inhibition of SLC7A11 or GPX4 activity using ferroptosis inducers resulted in enhanced sensitivity of tumor cells to radiotherapy [116].

Currently, there are few studies on ferroptosis combined with radiotherapy in the treatment of pancreatic cancer, but it suggests that radiotherapy combined with ferroptosis inducible drugs may provide a new method for the treatment of advanced, recurrent pancreatic cancer.

## 6. Conclusions

Research on programmed cell death and its molecular mechanisms has been increasing in recent years, and studies on ferroptosis and PDAC are rapidly developing. With the study of ferroptosis, its unique molecular mechanisms and roles in tumors are gradually emerging. Since pancreatic cancer is resistant to all current clinical treatment options, targeting the ferroptotic pathway may provide a good alternative approach to treat this deadly disease. Various preclinical studies have shown that certain drugs, such as artesunate and zalcitabine, can inhibit the development of PDAC by inducing ferroptosis, although these drugs still have off-target effects. The ultimate goal of the study is to develop clinically available drugs for the induction of the ferroptosis pathway that can cure PDAC alone or in combination with other drugs. Ferroptosis is an effective therapy for PDAC, but there is a lack of understanding of its mechanisms. In-depth and extensive basic research can help to develop more efficient treatment options.

## Figures and Tables

**Table 1:** Main ferroptosis inducers.

Target	Inducers	Function	Reference
System xc^-^	Erastin	Inhibits the activity system xc^-^ and affects the synthesis of GSH	[17]
	Glutamate	Inhibits the activity of system xc^-^, high extracellular glutamate concentrations prevent cystine import.	[26]
	Sorafenib	Inhibits the activity of system xc^-^ (directly affects the synthesis of GSH in a narrow concentration range)	[26]
GPX4	RSL3	Covalently binds to the selenocysteine residue of GPX4	[27]
	Cisplatin	Binds to GSH to inactivate GXP4	[33]
	FIN56	Induces GPX4 degradation; Binds and activates SQS to deplete CoQ10	[35]
	FINO2	Indirectly inhibit GPX4 activity, oxidize labile iron	[36]

**Table 2:** Main ferroptosis inhibitors.

Target	Inhibitors	Function	Reference
Iron chelator	Deferoxamine	Suppress ROS accumulation	[17]
	Ciclopirox olamine	Suppress ROS accumulation	[17]
	Thymosin β4	Enhance anti-oxidative processes	[37]
Cystine uptake	β-ME	Increases the uptake of cystine to improve the activity of GPX4	[17]
LPO	Ferrostain-1	Inhibit lipid PUFAs, block lipid peroxidation	[28]
	Lipoxstrain-1	Inhibit lipid peroxidation	[29]
	N-Acetylcysteine	Neutralizes toxic lipids generated by ALOX5	[30]

## Data Availability

Not applicable.
